# Untargeted Metabolomic Analysis of Rat Neuroblastoma Cells as a Model System to Study the Biochemical Effects of the Acute Administration of Methamphetamine

**DOI:** 10.3390/metabo8020038

**Published:** 2018-06-07

**Authors:** Garth L. Maker, Tobias Green, Ian Mullaney, Robert D. Trengove

**Affiliations:** 1 School of Veterinary and Life Sciences, Murdoch University, Perth, WA 6150, Australia; tobiasgreen91@gmail.com (T.G.); I.Mullaney@murdoch.edu.au (I.M.); 2 Separation Science and Metabolomics Laboratory, Murdoch University, Perth, WA 6150, Australia; R.Trengove@murdoch.edu.au

**Keywords:** B50 cell line, glutamate, GC–MS, methamphetamine, neurotoxicity

## Abstract

Methamphetamine is an illicit psychostimulant drug that is linked to a number of diseases of the nervous system. The downstream biochemical effects of its primary mechanisms are not well understood, and the objective of this study was to investigate whether untargeted metabolomic analysis of an in vitro model could generate data relevant to what is already known about this drug. Rat B50 neuroblastoma cells were treated with 1 mM methamphetamine for 48 h, and both intracellular and extracellular metabolites were profiled using gas chromatography–mass spectrometry. Principal component analysis of the data identified 35 metabolites that contributed most to the difference in metabolite profiles. Of these metabolites, the most notable changes were in amino acids, with significant increases observed in glutamate, aspartate and methionine, and decreases in phenylalanine and serine. The data demonstrated that glutamate release and, subsequently, excitotoxicity and oxidative stress were important in the response of the neuronal cell to methamphetamine. Following this, the cells appeared to engage amino acid-based mechanisms to reduce glutamate levels. The potential of untargeted metabolomic analysis has been highlighted, as it has generated biochemically relevant data and identified pathways significantly affected by methamphetamine. This combination of technologies has clear uses as a model for the study of neuronal toxicology.

## 1. Introduction

Methamphetamine (CAS 537-46-2), also known as speed or ice, is an illicit psychostimulant drug that is increasingly being abused around the world. In Australia, it is estimated that 7% of people over the age of 14 have used methamphetamine at least once [1]. Methamphetamine is highly addictive, and long-term users develop both dependence and tolerance. It is a proven cause of violent and psychotic behaviour [2], and is associated with diseases of both the cardiovascular and nervous systems, such as schizophrenia, Parkinson’s disease and depression [3,4,5,6]. 

The effects associated with methamphetamine use are primarily due to its effect on levels of monoamine neurotransmitters, including dopamine, noradrenaline and serotonin [7]. Methamphetamine enters the presynaptic cleft and, subsequently, the presynaptic vesicles, where it causes a change in pH [8,9]. This results in an increased release of monoamines into the cytosol and then the synapse [10]. Of all monoamines, dopamine plays the key role in methamphetamine-induced toxicity. Elevated dopamine levels lead to the formation of dopamine quinones, which bind to cysteine residues, commonly found in the active sites of enzymes [11], blocking the function of these enzymes [12,13,14]. It also blocks the effect of several antioxidants, including glutathione and superoxide dismutase (EC 1.15.1.1) [12,14,15,16], leading to a build-up of reactive oxygen species (ROS), which can cause damage to DNA, RNA, proteins and lipids [17], and neuronal apoptosis [18].

A second key neurotoxic mechanism of methamphetamine is via release of glutamate, the primary excitatory neurotransmitter in the brain [19]. Briefly, methamphetamine causes the release of dopamine and the activation of the dopamine D1 receptor, which stimulates the γ-aminobutyric acid (GABA)-ergic pathway and activates GABA-A receptors. This promotes the release of glutamate from the neurons, where they trigger excessive activation of the *N*-methyl-d-aspartate (NMDA) receptor, causing excitotoxicity [20,21]. The activated NMDA receptor allows for the influx of calcium ions into the neuron, which leads to the generation of ROS, causing oxidative stress, and nitric oxide, which can further react with ROS to form the neurotoxin, peroxynitrate [22]. It has also been demonstrated that glutamate and dopamine can interact to increase this toxicity [23]. 

It is clear that the combined toxicities of dopamine and glutamate have a substantial impact on the neuronal cell, however, the resultant effects on the biochemistry of the cell are less well-understood. Studies have observed changes in the metabolism of different parts of the brain following methamphetamine use [24,25], meaning that metabolomic analysis, the study of small molecules produced and consumed during cellular metabolism, may provide the ability to profile such changes in considerable detail. While there have been extensive studies on gene expression following methamphetamine treatment [26,27,28], there have been relatively few studies applying metabolomic technologies to date. Shima et al. [28] dosed rats with methamphetamine and profiled metabolites in both urine and plasma, observing that methamphetamine appeared to inhibit glycolysis, the TCA cycle and β-oxidation of fatty acids. McClay et al. [29] studied changes to brain tissue in dosed rats, observing an initial increase in energy metabolism, followed by the breakdown of mitochondria and increased antioxidants. Zaitsu et al. [30] profiled metabolites in the urine and plasma of methamphetamine-addicted rats, and identified changes, primarily in fatty acids, while Choi et al. [31] observed similar changes in the hair of a rat self-administration model. Zheng et al. [32] conducted a dose escalation study on rats and observed an increased TCA cycle and lipid metabolism, along with an increased stimulation and decreased inhibition of the nervous system. 

As seen in the above studies, the application of metabolomics to toxicological studies has largely been through animal studies. Within the toxicology community, there continues to be considerable emphasis on identifying alternative approaches that avoid the use of animals. In vitro models for assessing toxicity using metabolomics have been developed, but have primarily focused on hepatotoxicity [33,34]. Very few studies have used this approach for the assessment of neurotoxicity [35]. We have recently demonstrated that gas chromatography–mass spectrometry (GC–MS) metabolomic analysis could be used to generate meaningful biochemical data about the effects of a high dose of insecticides on a continuous cell line, the rat B50 neuroblastoma [36]. The aim of this study was therefore to determine whether this same model could be applied to the study of methamphetamine toxicity, and to generate biologically relevant data.

## 2. Results

**Figure 1:**
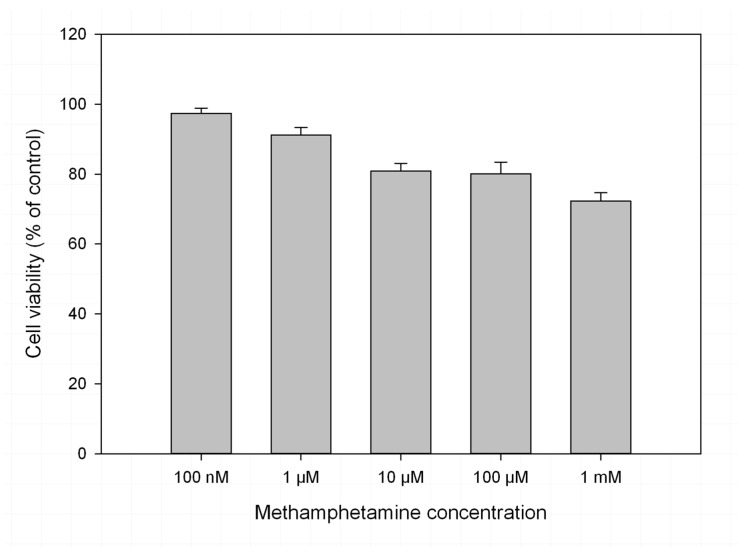
Cell viability of rat B50 neuroblastoma cells untreated or treated for 48 h with methamphetamine at 100 nM, 1 μM, 10 μM, 100 μM and 1 mM, determined using cell count with Trypan Blue exclusion. n = 3 for all conditions and error bars are SEM.

Cell viability following treatment with methamphetamine at different doses was assessed using cell counting with Trypan blue exclusion (Figure 1). Treatment with 1 mM methamphetamine for 48 h caused cell viability to drop to 61.3 ± 4.4%, while treatment with 10 and 100 mM caused decreases to 79.1 ± 3.2% and 74.1 ± 4.3%, respectively. Lower doses caused decreases in viability of less than 10%. As more than 50% of cells remained viable at 1 mM methamphetamine, this dose was used for subsequent experimentation.

**Figure 2:**
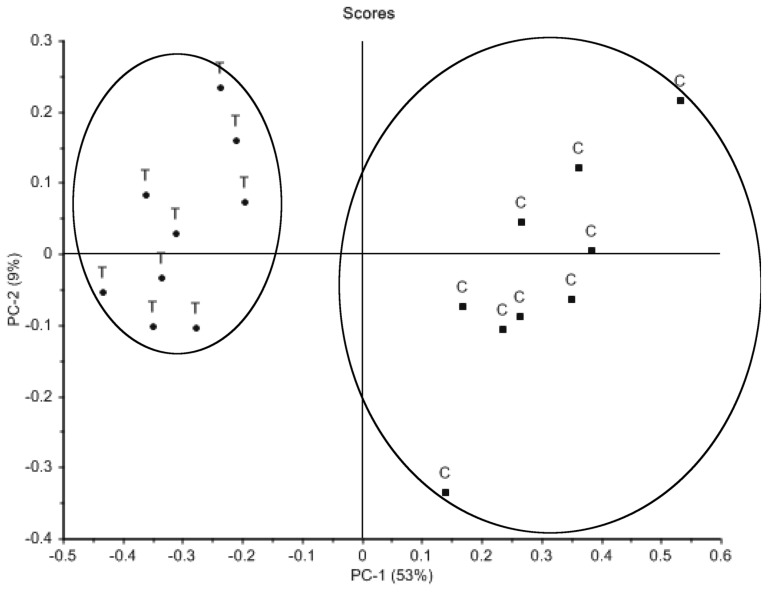
Principal component analysis scores plot of metabolite profiles generated by GC–MS analysis of rat B50 neuroblastoma cells (intracellular samples) untreated (C) (n = 9) or treated with 1 mM methamphetamine for 48 h (T) (n = 9).

**Figure 3:**
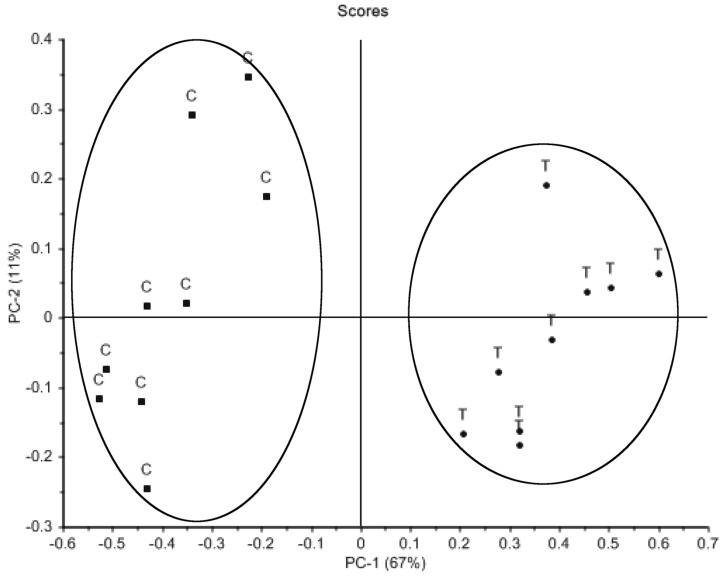
Principal component analysis scores plot of metabolite profiles generated by GC–MS analysis of culture medium (extracellular samples) from rat B50 neuroblastoma cells untreated (C) (n = 9) or treated with 1 mM methamphetamine for 48 h (T) (n = 9).

Principal component analysis of the intracellular metabolite profiles of methamphetamine-treated cells (Figure 2) showed a clear distinction between control and treated cells, with the majority of the variance between the two groups explained by PC-1 (53%). The PCA of the medium taken from these cells (extracellular) (Figure 3) also showed two distinct groups, with PC-1 again explaining the majority of the variance (67%). PCA loadings identified 35 metabolites, which contributed most to the variance observed between methamphetamine-treated and untreated cells (X-loading > 0.1), and 49 metabolites in the corresponding extracellular samples. Further analysis focused on the 35 metabolites identified in the intracellular samples (Table 1), allowing for the comparison of relative intracellular and extracellular changes. 

### 2.1. Amino Acids

The main overall trend observed was a 1.8-fold intracellular increase in amino acid levels in methamphetamine-treated cells, and a 1.6-fold extracellular increase. Six amino acids showed significant changes in both intracellular and extracellular samples. Of particular interest is the 3.3-fold intracellular increase in glutamate, coupled with a 2.1-fold extracellular increase (both *p* < 0.01). *N*-acetylglutamate levels also showed a 4.7-fold intracellular increase (*p* < 0.01) and a 1.4-fold extracellular increase (*p* < 0.05), a further indicator of increased glutamate levels. Aspartate levels showed an 8.0-fold intracellular increase and a 2.6-fold extracellular increase (both *p* < 0.01), while methionine showed a 3.5-fold intracellular increase and a 2.5-fold extracellular increase (both *p* < 0.01). A 2.3-fold intracellular decrease (*p* < 0.01) was observed in levels of phenylalanine, while serine levels showed a 3.1-fold intracellular decrease (*p* < 0.01) and a 3.0-fold extracellular increase (*p* < 0.01). 

Intracellular proline levels decreased 1.4-fold (*p* < 0.01), and a non-significant change was observed in alanine levels, with a 1.3-fold intracellular decrease, although a 1.4-fold extracellular increase was significant (*p* < 0.01). Intracellular leucine levels did increase 1.4-fold, although this was not significant. A number of other amino acids showed significant extracellular increases in methamphetamine-treated cells, namely, arginine, isoleucine, threonine, tryptophan, tyrosine and valine (all *p* < 0.01, except arginine and threonine, where *p* < 0.05).

### 2.2. Other Metabolites

Several other metabolites were also affected by methamphetamine treatment. Intracellular γ-amino-butyric acid (GABA) decreased 3.4-fold, which was a significant difference (*p* < 0.01), but it was not detected in the medium. The only significant changes observed in carbohydrates were a 1.1-fold intracellular increase in galactose, coupled with a 1.2-fold extracellular increase, and a 1.6-fold intracellular increase in an unidentified carbohydrate (all *p* < 0.01). Other notable, non-significant, intracellular changes observed were in glucose, which was 2.4-fold higher, and mannose, which was 1.6-fold lower (both *p* > 0.05). Metabolites that showed intracellular increases include fumarate (2.1-fold, *p* < 0.01), succinate (1.6-fold, *p* < 0.05) and pyroglutamate (1.7-fold, *p* < 0.01). A significant intracellular increase in octadecanoate (1.3-fold, *p* < 0.05) was observed, along with a non-significant extracellular decrease (1.2-fold). Intracellular cholesterol levels decreased 1.8-fold (*p* < 0.01) and intracellular glycerol levels also decreased (4.2-fold, *p* < 0.01). Tryptamine, a neuromodulator that promotes the release of serotonin from neurons, showed a 2.1-fold intracellular increase (*p* < 0.01).

## 3. Discussion

This study has successfully observed differences in the intracellular metabolite profile of cultured neuronal B50 cells and, as can be seen below, associated specific differences with known effects of methamphetamine exposure. Untargeted metabolomic analysis detected hundreds of individual metabolite features and PCA showed separate grouping of the treatment and control. These data, comprising metabolites from a range of classes, highlight the suitability of combining cultured cells with metabolomics for the monitoring of cellular responses to toxic chemical exposure. This has clear potential for future toxicology testing practices, particularly given the increasing interest in using human-derived cell lines [37]. Such an approach, for the prediction of toxic effects on biochemical pathways, is not only essential for enhancing our understanding of toxicology, but is also essential for the reduction of the use of animals in toxic chemical testing. As previously advocated, a ‘whole of cell’ metabolomic approach has benefits over individual biomarkers derived from animal studies, because it allows changes across entire biochemical pathways to be profiled, giving a more complete picture of the effects of toxins [36]. Future studies should compare the metabolic effect of methamphetamine on a human neuronal cell line with that on the rat B50 neuronal cell line.

The changes profiled in the metabolites of cells exposed to methamphetamine are interpretable in the context of the known biochemical effects of methamphetamine exposure, as demonstrated below, with a focus on amino acids and energy metabolism.

### 3.1. Amino Acids

Significant increases were observed in both intracellular and extracellular levels of glutamate. Methamphetamine stimulates the release of glutamate via dopamine and GABA [19], which causes the activation of NMDA receptors and, subsequently, excitotoxicity. This leads to oxidative stress and further damage to the neuronal cell [20,21]. In terms of the metabolites identified as important in this study, it is clear that glutamate had a substantial influence on the cells. Increased glutamate levels were previously observed in a rat model of methamphetamine administration [32]. The mechanism described would have also contributed to an decrease observed in levels of the neurotransmitter, GABA. GABA is oxidised to both succinate and glutamate, both of which showed a significant intracellular increase. The oxidation of GABA primarily occurs when glucose is limited, as succinate can provide an alternative carbon source, suggesting that glucose transport was interrupted. The importance of GABA in response to methamphetamine has recently been reviewed by Chiamulera et al. [38].

The expected excitotoxic effect, and the cell’s need for protection against it, appears to have driven other changes observed in the amino acid levels. The significant increase in aspartate levels suggests that the cells may be attempting to reduce glutamate and the associated excitotoxicity via aspartate transaminase (AST), which converts glutamate into α-ketoglutarate, while producing aspartate from oxaloacetate. Increased aspartate levels were also observed in the aforementioned rat study [32]. Serine has been demonstrated to have both antioxidant and neuroprotective effects [39,40], and thus may have been produced by the cell in the early response to exposure and then transported out in a sampling time of 48 h. Methionine, which is also known to have antioxidant properties [41], showed both intracellular and extracellular increases. 

In terms of these significant changes in amino acid levels, the general mechanism suggested by the metabolomic data is that methamphetamine treatment causes the production and release of glutamate, which the cell attempts to balance by increasing the production of aspartate via AST. The oxidative stress caused by glutamate stimulates the production of methionine and serine. The intracellular decrease in serine suggests that it is produced during the initial stages of methamphetamine exposure, and methionine is produced later. 

### 3.2. Energy Metabolism

Observed changes in carbohydrate levels were less pronounced than in amino acids. The high levels of glucose present in DMEM (4.5 g/L) could mask changes in carbohydrate metabolism. Glucose is transported into neuronal cells via the specific GLUT3 transporter [42], and recent work has shown that methamphetamine can inhibit GLUT3, preventing the transport of glucose into the neuron, especially at high doses [43]. It was therefore expected that glucose levels would be depleted within the cells. However, it is likely that by the time cellular metabolism was quenched at 48 h, methamphetamine had been degraded, allowing for the resumption of glucose transport via GLUT3. The inclusion of additional time points in future studies would help to clarify the effect on glucose levels.

Methamphetamine is known to trigger the production of reactive oxygen species, via dopamine quinone production [44], which causes damage within the cell. This damage may affect the ability of the cell to utilise the glucose that it transports in after an extended period of exposure to methamphetamine, further contributing to build-up within the cell. The observed intracellular increases in fumarate and succinate also indicate that aerobic metabolism was affected by treatment. Pyroglutamate is an uncommon amino acid that can be produced during oxidative stress [45] and has been shown to interfere with energy production in a rat cerebral cortex [46]. 

Previous studies on the biochemical effects of methamphetamine treatment have indicated that energy metabolism is one of the most commonly affected pathways. Shima et al. [28] observed that methamphetamine appears to inhibit glycolysis, the TCA cycle and β-oxidation of fatty acids in rats 24 h after exposure. By 96 h, the urine and plasma metabolite profiles of control and treated rats were indistinguishable, indicating that changes in energy metabolism likely occur during the period immediately following exposure. McClay et al. [29] studied mouse brain tissue 1 h after methamphetamine exposure, either once or daily for up to 5 days. This study found that an acute dose perturbed energy metabolism, including the TCA cycle, while repeated doses led to mitochondrial damage, disrupting energy generation. Zheng et al. [32] observed energy metabolism increases corresponding to escalating doses in rats, specifically, increases in the TCA cycle and lipid metabolism. Samples in this study were collected only 1 h after administration and thus reflect an acute effect of the drug. 

The present data indicate that carbohydrate metabolism was affected by methamphetamine treatment, although the specific pathways involved are unclear. This highlights an important consideration in metabolomic studies. The time of the sampling, as a single time point, can only provide a snapshot of the biochemical status of the cell. In order to fully understand the effect of methamphetamine treatment on carbohydrate metabolism in neuronal cells, a time course should be conducted over the course of treatment. Further refinements to the approach would include the use of pooled quality control samples, which are routinely employed in metabolomic studies, and would allow the biological variance within the dataset to be fully separated from the instrumental variance.

### 3.3. Conclusions

Metabolite profiles of methamphetamine-treated cells and corresponding medium samples showed changes that fit with what is known about the effects of this drug on neuronal cells. Most notably, the data demonstrated that glutamate release and subsequent excitotoxicity and oxidative stress constituted a major part of the response of the neuronal cell to methamphetamine. Following this, the cells appeared to engage a number of amino acid-based mechanisms to attempt to reduce glutamate levels. This likely included the well-characterized use of aspartate transaminase to convert glutamate into aspartate, leading to a marked increase in aspartate levels. The data also indicate that two mechanisms not previously linked to methamphetamine treatment may be involved in the response of the cells, namely the production of serine and methionine as antioxidants. Expected changes to energy metabolism were not clear from the data, possibly because these changes occur over a shorter timeframe than the 48-hour timeframe used in the current study. 

Untargeted metabolomic analysis identified multiple biochemical pathways in the B50 neuroblastoma cell that were affected by methamphetamine treatment. The data generated were biochemically relevant and show the potential of this technology in studying the response of cells to drugs and other toxins. By expanding the use of metabolomic technologies in toxicology analysis, it will be possible to identify links between biochemical pathways and highlight pathways not previously known to be involved in the cellular response, which may provide new targets for the development of protective therapies.

## 4. Materials and Methods

### 4.1. Chemicals

All chemicals were purchased from Sigma Aldrich (Sydney, Australia) in the highest purity available, unless otherwise stated. Solvents were purchased from LabScan (Seacliff, Australia) in the highest purity available.

### 4.2. Cell Culture

B50 rat neuroblastoma cells were obtained from the European Cell Culture Association via Sigma Aldrich. Cells were maintained at 37 °C and 5% CO_2_ in Dulbecco’s modified Eagle medium (DMEM) and supplemented with 1% glutamine, 1% penicillin/streptomycin and 5% fetal calf serum (heat-treated at 56 °C for 2 h). Passages between 4 and 6 were used for experimentation. Prior to treatment, cells were cultured in 6-well plates until 75–85% confluent. Methamphetamine exposure was conducted in triplicate on cells from three consecutive passages (n = 9 for each condition). For each of the treatments, 3 plates were exposed to 1 mM methamphetamine (in sterile water) for 48 h, while another 3 were left untreated as control (sterile water vehicle added).

In order to investigate the biochemical effects of methamphetamine exposure, the dose of methamphetamine used needed to leave a sufficient number of cells alive to provide a metabolism that could be profiled. A 1 mM dose of methamphetamine has previously been shown to decrease cell viability by up to 50% [47,48] and similar doses have been used in other in vitro studies of methamphetamine toxicity, with exposures from 24 to 48 h [49,50]. For this reason, cells were exposed in triplicate to methamphetamine at the following doses for 48 h: 100 nM, 1 μM, 10 μM, 100 μM and 1 mM. Cell viability was determined using Trypan blue staining. The medium was removed and 500 μL of trypsin (EC 3.4.21.4) was added. The plates were returned to the incubator for 5 min and then 2 mL of DMEM was added to terminate trypsinization. A 1 mL aliquot of the cell suspension was centrifuged at 4500× *g* for 5 min, and the supernatant was discarded. The resultant pellet was resuspended in 1 mL of PBS. The cell suspension was mixed at a ratio of 1:1 with 0.4% Trypan Blue and counted using an Improved Neubauer Haemocytometer (Sigma Aldrich) and an Olympus CKX41 inverted light microscope (Olympus, Mt. Waverley, Australia). Cells were counted within the four corner squares; cells falling on the centre borders were counted, but those outside the borders were excluded. An average of two counts was used to represent a mean cell count for that plate. The viable (unstained) and non-viable (blue-stained) cells were counted separately and used to estimate viability.

### 4.3. Sample Preparation

Optimization of the method indicated that a single well on a 6-well plate did not contain sufficient cells (approximately 2 × 10^6^ cells per well) to yield adequate metabolites for reproducible GC–MS profiling (data not shown). For this reason, 5 wells from each 6-well plate were harvested together, representing approximately 10^7^ cells. 

After 48 h incubation, the plates were removed from the incubator and placed on ice. A 40 μL aliquot of the culture medium was removed, and 5 wells were combined in a 1.5 mL tube that was immediately placed on dry ice. The remaining medium was removed from each well and the cells were carefully washed with 1 mL of phosphate-buffered saline (PBS) solution at 4 °C. This wash was discarded and 100 μL of PBS was added to each well. Recent studies [51] have shown that cold PBS is the best solution for quenching metabolism in cultured mammalian cells. The cells from 5 wells of each 6-well plate were harvested using a 16 mm cell scraper and immediately placed on dry ice. Both the cell and medium samples were freeze-dried using a Freezone 2.5 Plus (Labconco, Kansas City, MI, USA). The remaining well on each 6-well plate was used to provide a mean cell count using Trypan blue staining, as described above.

Following freeze-drying, samples were centrifuged at 5000× *g* for 5 min. Metabolites were extracted using 500 μL methanol containing ^13^C_6_-sorbitol, as an internal standard, at a concentration of 3 μg/mL. Methanol extraction has been demonstrated to be the optimal solvent for the extraction of adherent cells for GC–MS analysis [29]. Solutions were transferred to fresh tubes and lysed in a Procellys tissue lyser (Bertin Corporation, Rockville, MD, USA) at 6500 rpm for 2 × 20 s. Samples were subsequently centrifuged at 13,200× *g* for 5 min, and 400 μL of the supernatant was transferred to a fresh tube. The volume was reduced using a Concentrator Plus rotary vacuum concentration (Eppendorf South Pacific, North Ryde, Australia), and 250 μL water was added to facilitate freezing at −80 °C. Once frozen, samples were freeze-dried.

Dried samples were derivatised according an established protocol [52,53]. Methoxyamine (20 mg/mL in pyridine) was added (20 μL), and the samples were mixed in a Thermomixer Comfort (Eppendorf South Pacific) at 1200 rpm for 90 min at 30 °C. The samples were centrifuged at 13,200× *g* for 1 min, and 40 μL of *N*-Methyl-*N*-(trimethylsilyl) trifluoroacetamide (MSTFA) was added. The samples were returned to the thermomixer and mixed at 300 rpm for 30 min at 37 °C. Once derivatised, the samples were transferred to 2 mL GC vials, and 5 μL of alkanes (C_10_-C_36_, 0.625 mg/mL in hexane) was added to allow for the calculation of a Kovat’s index.

### 4.4. Analysis

Samples were analysed using an Agilent 6890 gas chromatograph, with an Agilent 7863 autosampler, coupled with an Agilent 5973N mass spectrometer (Agilent Technologies Australia, Mulgrave, Australia). Helium was used as a carrier gas, and the column was an Agilent Factor Four VF-5 ms fused silica capillary column (dimensions 30 m × ID = 0.25 mm × Df = 0.25 μm + 10 m EZ-Guard). Using an established method [52,53], the temperature of the inlet was set to 230 °C, and the initial oven temperature was set to 70 °C. Temperature was increased at 1 °C/min for 6 min and then ramped up by 5.63 °C/min to a final temperature of 330 °C, and held for 10 min. The transfer line to the mass spectrometer was set to 330 °C and the ion source, to 230 °C and 70 eV, with a solvent delay of 8.0 min. The detector was set to full scan, monitoring a mass range of *m*/*z* 45–600 at 1 scan per second. GC–MS data were deconvoluted using AnalyzerPro v. 2.7.0 (SpectralWorks, Runcorn, UK), normalized to viable cell count and referenced against an in-house target component library, which comprised both mass spectrum and retention index.

### 4.5. Statistical Analysis

The results of GC–MS analysis were exported to The Unscrambler X v. 10.1 (CAMO Software, Oslo, Norway), and log_10_ was transformed. Principal component analysis (PCA) was undertaken, using a non-linear iterative partial least squares algorithm, cross validation and no rotation. Data from PCA were used to identify those metabolites that contributed the most to the variance observed between methamphetamine-treated cells and control. Subsequent statistical analysis was undertaken using SPSS Statistics v. 21 (IBM Corporation, Armonk, NY, USA). Metabolite levels in control and treated samples were compared using an unpaired Student’s *t*-test, assuming equal variance, coupled with the Benjamini-Hochberg false discovery rate correction. All error bars represent the standard error of the mean (SEM).

## Figures and Tables

**Table 1:** Metabolites identified by PCA as contributing most to the variance between untreated and methamphetamine-treated rat B50 neuroblastoma cells, and the fold change observed in the treated cells and medium (n = 9), compared to the untreated ones (n = 9). ‘-’ indicates no change. Statistical significance was determined using Student’s *t*-test and is indicated as * *p* < 0.05; ** *p* < 0.01.

Metabolite	Cells (Fold Change)	Medium (Fold Change)
Amino Acids		
*N*-Acetylglutamate	4.7 **	1.4 *
l-Alanine	0.8	1.4 **
l-Arginine	1.1	1.3 *
l-Aspartate	8.0 **	2.6 **
l-Glutamate	3.3 **	2.1 **
Glycine	-	1.1
l-Isoleucine	0.9	1.7 **
l-Leucine	1.4	1.1
l-Methionine	3.5 **	2.5 **
l-Phenylalanine	0.5 **	1.9 **
l-Proline	0.7 **	1.1
Pyroglutamate	1.1	1.7 **
l-Serine	0.3 **	3.0 **
l-Threonine	0.9	1.3 *
l-Tryptophan	-	1.7 **
l-Tyrosine	-	1.7 **
l-Valine	-	1.4 **
Carbohydrates		
Arabitol	-	-
Fructose	1.2	-
Galactose	1.1 **	1.2 **
Glucose	2.4	-
Mannose	0.6	-
Unidentified carbohydrate 1	1.6 **	1.2
Unidentified carbohydrate 2	1.3	0.9
Unidentified carbohydrate 3	1.3	1.1
Other metabolites		
Cholesterol	0.6 **	not detected
Erythronate	0.9	1.1
Fumarate	2.1 **	1.1
GABA	0.2 **	not detected
Glycerol	0.2 **	-
Hexadecanoate	-	1.1
myo-Inositol	1.3 **	-
Octadecanoate	1.3 *	0.8
Succinate	1.6 *	-
Tryptamine	2.1 **	not detected
